# Investigating the Efficacy of Layered Moderate Tension Reduction Suturing in Facial Aesthetic Surgery

**DOI:** 10.7759/cureus.85000

**Published:** 2025-05-28

**Authors:** Gui H Wang, Jin Y Gang, Yan Li

**Affiliations:** 1 Plastic Surgery, Tongling Municipal Hospital, Anhui Medical University, Tongling, CHN; 2 Plastic Surgery, Wuxi Kaiyi Hospital, Wuxi, CHN; 3 Otorhinolaryngology, Xianghe County People's Hospital, Langfang, CHN; 4 Otorhinolaryngology, Shanghai Yida Hospital, Shanghai, CHN; 5 Otorhinolaryngology, Wuxi Huawei Hospital, Wuxi, CHN

**Keywords:** cosmetic and plastic surgery, head and facial region, layer-by-layer moderate tension reduction suture technique, patient and observer scar assessment scale (posas), scar formation, vancouver scar scale (vss)

## Abstract

Objective: This study aims to investigate the efficacy of the layer-by-layer moderate tension reduction suture technique in head and facial aesthetic plastic surgery.

Methods: A retrospective analysis was performed on the clinical data of 80 patients who underwent head and facial cosmetic and plastic surgery in the outpatient department of our hospital from April 2022 to April 2024. Among these, the experimental group received the layer-by-layer moderate tension reduction suture technique, whereas the control group received the traditional suture technique. The incidence of surgical complications, scar width, and scar quality metrics derived from the Patient and Observer Scar Assessment Scale (POSAS) and Vancouver Scar Scale (VSS) scores were compared between the two groups.

Results: The experimental group had a longer operation time but no complications (0%), compared to the control group's 17.5% complication rate. The χ² test confirmed the experimental group's significantly lower complication rate (P < 0.05). At one, three, six, and 12 months postoperatively, the experimental group had significantly smaller scar widths, lower POSAS scores, and lower VSS scores compared to the control group (all P < 0.05).

Conclusion: The layer-by-layer moderate tension reduction suture technique demonstrated substantial advantages in head and facial aesthetic plastic surgery. It effectively minimized surgical complications, reduced scar width, and enhanced patients' scar appearance scores, making it highly worthy of clinical promotion.

## Introduction

The head and face are the most exposed areas of the human body and are subject to the highest aesthetic standards [1-4]. In head and facial cosmetic and reconstructive surgery, suture techniques play a critical role in determining the quality of postoperative incision healing, scar appearance, and overall cosmetic outcomes [5]. Traditional suture methods may have limitations, such as causing excessive tension at the incision site, which can result in widened or hypertrophic scars. The layer-by-layer moderate tension reduction suture technique aims to systematically reduce tension across all layers of the head and face tissues, thereby optimizing postoperative results to the greatest extent possible. This technique has garnered significant attention in the field of cosmetic and plastic surgery in recent years [6]. This study aims to thoroughly investigate the application value of this technique in head and facial cosmetic and reconstructive surgery, providing robust evidence for clinical practice.

## Materials and methods

General information

A retrospective analysis was performed on the clinical data of 80 patients who underwent head and facial cosmetic or reconstructive surgery at our hospital's outpatient department between April 2022 and April 2024. Among these, the experimental group (n = 40; 13 males, 27 females) received the layer-by-layer moderate tension reduction suture technique. Their age ranged from 21 to 56 years (mean ± standard deviation (SD): 29.95 ± 5.39 years), with an incision length of 30.80 ± 24.16 cm. The control group (n = 40; 14 males, 26 females) underwent traditional suture techniques. Their age also ranged from 21 to 56 years (mean ± SD: 30.10 ± 4.67 years), with an incision length of 32.45 ± 18.4 cm. No statistically significant differences were observed in age, gender, or incision length between the two groups (P > 0.05), ensuring comparability. This study was approved by our hospital’s ethics committee (the Medical Ethics Committee of Tongling Municipal Hospital; approval no.: 20220401), and all participants provided informed consent prior to enrollment.

The inclusion criteria for this study were as follows: First, patients scheduled to undergo head and facial cosmetic or reconstructive surgery, including procedures such as facial trauma debridement and suturing, scar revision, and wound closure following tumor excision. Second, patients with high aesthetic expectations regarding postoperative incision outcomes, such as performers or individuals who are highly concerned about their appearance. Third, patients aged between 18 and 65 years, a range characterized by relatively stable physiological functions and optimal skin healing capacity. Fourth, patients without severe underlying medical conditions, such as uncontrolled diabetes, coagulopathy, advanced malignancies, or severe cardiovascular/cerebrovascular diseases. Fifth, patients with good psychological health who are capable of understanding surgical risks and actively participating in postoperative care and follow-up evaluations.

The exclusion criteria were as follows: First, emergency trauma patients with heavily contaminated wounds, complex tissue injuries, or associated compound injuries, which preclude the application of standard layered tension-reducing suture techniques. Second, patients who have undergone multiple surgeries in the same area, leading to altered local anatomy, significant tissue adhesions, or severe scar contractures. Third, patients with active facial infections, such as cellulitis or furuncles, where surgery could exacerbate infection and compromise healing. Fourth, patients with unstable mental health or allergies to suture materials, which may interfere with the surgical process or postoperative outcome assessment.

Surgical method

Experimental Group (Layer-by-Layer Moderate Tension Reduction Suturing Technique)

**Figure 1:**
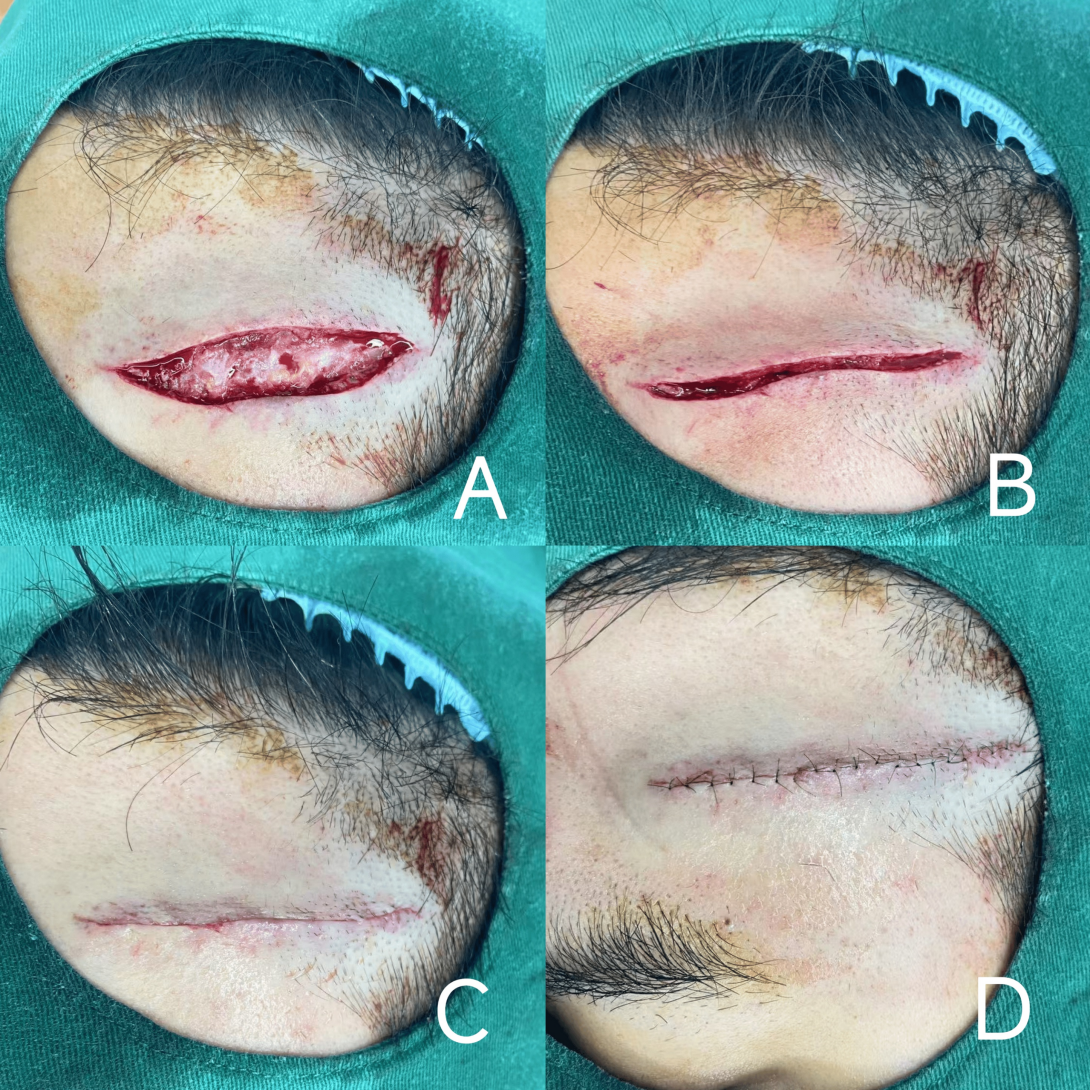
Layer-by-Layer Moderate Tension Reduction Suturing Technique

Deep tissue suturing (muscle and fascia layers): After completing the preoperative procedures on the head and face, meticulous suturing of the deep tissues was performed first. For the muscle layer, if there were large muscle lacerations, interrupted or mattress suturing was adopted. A round needle and relatively thick absorbable suture material were used, with the entry point approximately 0.5-1 cm away from the muscle edge. The stitch interval was determined based on the muscle thickness and tension, typically ranging from 1 to 2 cm. The fascia layer was usually sutured continuously or intermittently to ensure precise alignment. If fascial defects existed, patch repair was employed to restore structural integrity (Figures 1A-1B).

Subcutaneous tissue suturing: Appropriate absorbable sutures (e.g., fine polyglycolic-lactic acid (PGLA) sutures) were selected, and layered suturing was performed to minimize dead space formation. The angle of needle insertion was as parallel as possible to the subcutaneous tissue plane to avoid overly tight sutures that might compromise tissue viability. The stitch length was adjusted according to the thickness of the subcutaneous tissue, generally ranging from 0.5 to 1 cm. During the suturing process, the surrounding adipose tissue was gently drawn toward the center to ensure smooth and even subcutaneous tissue apposition, thereby promoting optimal skin adhesion (Figure 1C).

Skin suturing: When skin tension was relatively low, simple interrupted suturing was chosen. Small triangular needles and non-absorbable sutures (e.g., nylon thread) were used, with the needle entry point approximately 2-3 mm from the skin edge and a stitch interval of about 3-5 mm. When tying knots, moderate force was applied to ensure proper skin apposition without eversion or inversion. When skin tension was high, moderate tension-reducing suturing was required. Vertical mattress suturing or modified tension-reducing techniques were employed. For vertical mattress suturing, the needle penetrated through the dermis and part of the subcutaneous tissue. By adjusting the distance between the sutures on both sides of the skin edge, skin tension was effectively dispersed, reducing the risk of wound dehiscence (Figure 1D).

Control Group

Traditional suturing methods were followed, with sequential suturing of the deep, middle, and superficial tissue layers. Typically, the deep layer was simply sutured, while the middle and superficial layers were sutured in a relatively coarse manner, primarily focusing on wound closure with minimal attention to precise tension adjustment.

Observation indicators

Surgical-Related Indicators

The surgical incision length and operation time were recorded for both groups of patients.

Surgical Complications

The occurrence of postoperative complications, including infection, hematoma, and wound dehiscence, was recorded for both groups of patients, and the incidence rates were calculated.

Width of Scar Formation

The width of the widest part of the scar was measured at one, three, six, and 12 months postoperatively using high-precision measuring tools, with data accurate to 0.1 mm.

Patient and Observer Scar Assessment Scale (POSAS)

At one, three, six, and 12 months postoperatively, both patients and trained observers used the POSAS scale to evaluate scars. Patients rated subjective aspects such as pain, itching, color, and hardness, whereas observers assessed objective characteristics including vascular distribution, color, thickness, and softness. Total scores ranged from 0 to 44, with lower scores indicating better scar outcomes [7-12].

Vancouver Scar Scale (VSS)

The VSS scale was used to evaluate scars at one, three, six, and 12 months postoperatively. This scale assesses scar characteristics, including color, thickness, and vascular distribution, with total scores ranging from 0 to 15. Lower scores correspond to less noticeable scars [8].

Statistical analysis

Data were analyzed using IBM SPSS Statistics for Windows, Version 27 (Released 2021; IBM Corp., Armonk, New York, United States). Continuous data were presented as mean ± SD (x ± s), and comparisons between groups were performed using the independent-samples t-test. Categorical data were expressed as percentages (%), and intergroup comparisons were conducted using the χ² test. A two-tailed P-value < 0.05 was considered to indicate statistical significance.

## Results

Surgical-related indicators

There was no statistically significant difference in surgical incision length between the two groups, indicating comparability (P > 0.05). The experimental group had a significantly longer operation time than the control group (P < 0.01) (Tables 1-2).

**Table 1:** Incision Length Comparison Between Groups ((x ± s), mm)

Group	Incision length
Control group (n=40）	30.80±23.85
Observation group (n=40）	32.45±18.16
t-value	0.348
P-value	>0.05

**Table 2:** Operation Time Comparison Between Groups ((x ± s), min)

Groups	Duration of surgery
Control group (n=40)	67.70±26.40
Study group (n=40)	126.00±37.95
t-value	7.98
P-value	<0.001

Surgical complications

In the experimental group, no complications were observed (0%), while the control group had a complication rate of 17.5%. The χ² test showed the experimental group's complication rate was significantly lower (P < 0.05) (Table 3).

**Table 3:** Complication Comparison Between Groups (n, %)

Groups	Hematoma (n)	Infection (n)	Wound dehiscence (n)	Dead space formation (n)	Total incidence (%）
Control group (n=40)	1	1	2	2	15
Study group (n=40)	0	0	0	0	0
χ^2^ value	-	-	-	-	4.51
P-value	-	-	-	-	0.03

Width of scar formation

At one, three, six, and 12 months postoperatively, the experimental group exhibited significantly smaller scar widths compared to the control group (P < 0.05) (Table 4).

**Table 4:** Postoperative scar width comparison between groups (x ± s)

Groups	One month after surgery	Three months after surgery	Six months after surgery	12 months after surgery
Control group (n=40)	0.54±0.38	0.54±0.38	0.93±0.53	0.93±0.53
Study group (n=40)	0.05±0.02	0.05 ±0.02	0.09±0.03	0.09±0.03
t-value	8.242	8.242	9.985	9.985
P-value	<0.001	<0.001	<0.001	<0.001

POSAS

At one, three, six, and 12 months postoperatively, the experimental group demonstrated significantly lower POSAS scores compared to the control group (P < 0.05) (Table 5).

**Table 5:** POSAS Score Comparison Between Groups Post-surgery (x ± s) POSAS: Patient and Observer Scar Assessment Scale

Groups	One month after surgery	Three months after surgery	Six months after surgery	12 months after surgery
Control group (n=40)	16.75±7.07	16.75±7.07	9.2±6.25	7.5±5.08
Study group (n=40)	5.8±1.42	5.5 ±1.22	1.85±0.67	1.75±0.63
t-value	9.609	9.922	7.399	7.106
P-value	<0.001	<0.001	<0.001	<0.001

VSS

At one, three, six, and 12 months postoperatively, the VSS scores of the experimental group were significantly lower than those of the control group (P < 0.05) (Table 6).

**Table 6:** VSS Score Comparison Between Groups Post-surgery (x ± s) VSS: Vancouver Scar Scale

Groups	One month after surgery	Three months after surgery	Six months after surgery	12 months after surgery
Control group (n=40)	6.5±3.31	6.5±3.31	4.15±2.29	3.8±2.19
Study group (n=40)	2.8±0.69	2.75±0.63	1.75±0.63	0.9±0.30
t-value	6.932	7.049	6.382	8.31
P-value	<0.001	<0.001	<0.001	<0.001

## Discussion

Regarding surgical complications

The layer-by-layer moderate tension reduction suture technique, despite prolonging the operation time and requiring more precise handling, effectively alleviates tissue tension. This significantly reduces the incidence of surgical complications. In head and facial surgeries, excessive tension not only leads to local tissue ischemia and hypoxia but also creates an environment conducive to bacterial growth, thereby increasing the risk of infection. Moreover, it directly causes complications such as wound dehiscence and hematoma formation [13]. By applying the layer-by-layer tension reduction approach, each tissue layer heals under reduced tension, markedly lowering the occurrence of these complications.

Regarding scar formation width

Tension is a critical factor influencing scar width. In high-tension environments, fibroblast proliferation and disordered collagen fiber arrangement contribute to widened scars [14-16]. The layer-by-layer tension reduction suture technique progressively reduces tissue tension from deep to superficial layers, effectively minimizing the outward pulling force during scar healing. Consequently, scar width in the experimental group was consistently smaller than in the control group at all time points, demonstrating the technique's long-term efficacy in controlling scar width.

POSAS and VSS

The POSAS exhibited notable advantages in this study. This scale innovatively integrates patient self-assessment with observer evaluation, enabling a comprehensive assessment of postoperative outcomes. The patient self-assessment component encompasses subjective indicators such as pain and itching, as well as objective features including color, flexibility, thickness, and irregularity. These indicators not only reflect the patient's subjective experience after surgery but also directly correlate with their quality of life and satisfaction with the surgical outcome. Through this process, patients are empowered to participate in the evaluation of surgical results based on their intuitive experiences, thereby enhancing the comprehensiveness of the assessment and increasing patient engagement. The observer scoring component involves systematic evaluations by professionals across multiple dimensions, including vascular distribution, color, thickness, flexibility, and edge characteristics. Leveraging their extensive clinical experience and specialized knowledge, observers can provide more objective and precise judgments regarding the appearance and properties of postoperative tissues, offering a robust scientific basis for assessing surgical outcomes [17,18].

Additionally, the VSS played a pivotal role in this study. By quantitatively scoring four critical dimensions of scars - color, vascular distribution, thickness, and softness - the VSS clearly demonstrated the specific recovery conditions of scars under different suture techniques. For the layer-by-layer moderate tension-reducing suture technique, the application of VSS further validated its efficacy in inhibiting scar hyperplasia and improving scar texture, providing compelling evidence for the clinical value of this technique and aiding doctors in formulating more precise surgical plans [19].

In summary, through the utilization of the multi-dimensional and systematic assessment tools POSAS and VSS, this study conducted an in-depth analysis of the practical effects of the stepwise moderate tension suture technique in head and facial aesthetic plastic surgery, establishing a solid data foundation for its technical optimization and clinical promotion.

However, this study has certain limitations that warrant consideration. First, the relatively small sample size may limit the generalizability of the findings. Second, the limited follow-up duration restricts our ability to fully assess the long-term effects of the technique on scar formation and surgical outcomes. Future studies should aim to increase the sample size and extend the follow-up period to achieve more comprehensive and reliable research outcomes. Additionally, incorporating advanced imaging techniques or molecular markers could provide deeper insights into the mechanisms underlying the benefits of the layer-by-layer tension reduction suture technique.

## Conclusions

The application of the staged moderate tension reduction suture technique in head and facial aesthetic and plastic surgery has shown significant benefits. This technique not only effectively minimizes the incidence of surgical complications but also significantly reduces scar width, leading to superior scar quality scores based on evaluation criteria such as the POSAS and VSS scales. In summary, this technique demonstrates substantial clinical value for promotion in head and facial aesthetic and plastic surgery.
